# Italian validation of the workplace ostracism scale (WOS)

**DOI:** 10.3389/fpsyg.2025.1584118

**Published:** 2025-05-20

**Authors:** Daiana Colledani, Alessandro De Carlo, Rossella Falvo, Dora Capozza

**Affiliations:** ^1^Department of Psychology, Sapienza University of Rome, Rome, Italy; ^2^Department of Clinical and Experimental Medicine, University of Messina, Messina, Italy; ^3^Department of Philosophy, Sociology, Education, and Applied Psychology, University of Padova, Padova, Italy

**Keywords:** workplace ostracism, Italian WOS, nomological validity of the Italian WOS, workplace ostracism as a demand, job demands-resources theory, network analysis

## Abstract

**Introduction:**

The aim of this study was to validate the Italian version of the workplace ostracism scale (WOS), developed by Ferris and colleagues. Workplace ostracism (WO)—the perception of being ignored or excluded by colleagues or supervisors—is a painful experience that negatively impacts employees and the whole organization. We tested the unidimensional structure of the Italian WOS, its independence of social desirability issues, and invariance across genders and ages. We also tested the nomological validity of the WOS by considering ostracism as a job demand and including it in the job demands-resources (JD-R) theory.

**Method:**

A sample of Italian employees (*N* = 653), working for different organizations in several Italian regions, completed an online questionnaire. Data were analyzed using exploratory and confirmatory factor analyses. Network analysis was applied to test the nomological validity of the scale.

**Results:**

Findings confirmed the unifactor structure of the Italian WOS and its invariance. Social desirability only absorbed a limited portion of variance of ostracism items. Data also supported the nomological validity of the WOS, that is, the expected association of ostracism with basic need frustration, lower work engagement, altruism, and performance.

**Discussion:**

In the discussion, we clarified the advantages of conceiving ostracism as a job demand and using network analysis to verify the JD-R theory. Practical implications of findings in order to contain workplace ostracism were commented.

## 1 Introduction

Ostracism has been defined by Williams (2001) as the extent to which an individual perceives he/she is ignored or excluded by other people. Ostracism is a universal phenomenon that occurs across genders, ages, and cultures (Williams, 1997). Regarding organizational settings, it has been found that 66% of the employees interviewed had received the silent treatment over 5 years; 29% reported that other people left the room when they entered (Fox and Stallworth, 2005).

Ostracism is a painful experience: it activates the same brain areas that are activated by physical pain (Eisenberger et al., 2003). In addition, it is related to the frustration of four primary needs (see Williams, 2007, 2009, for reviews): need to belong (individuals are afraid of being excluded from the group); need for self-esteem (excluded individuals have the impression they have done something wrong); need for control (they believe their actions have no impact on others); and, finally, need for meaningful existence (excluded individuals perceive themselves as nonexistent or unworthy of attention).

Ostracism may cause aggressive behaviors (Twenge et al., 2001; see also van Beest et al., 2012; Poon and Wong, 2019) and may hinder prosocial behaviors (Twenge et al., 2007; see also Shao et al., 2023). Regarding cognitive processes, it may be associated with a deconstructive cognitive state (Baumeister, 1990): a defensive mental state, characterized by refusal to engage in interpretative thoughts and little concern for long-term goals (Twenge et al., 2003). Ostracism may, therefore, be related to risk-taking behaviors (e.g., Svetieva et al., 2016).

In the workplace (Ferris et al., 2008), ostracism is positively related to depression, anxiety, and turnover intentions. It is negatively related to job satisfaction and organizational citizenship behaviors (OCBs). The latter are prosocial behaviors not included in the formal work system, but useful for the effective functioning of the organization (see Organ, 1988). Therefore, ostracism negatively affects individual employees and the entire organization.

These negative effects have been explained by using the temporal need-threat model of ostracism (Williams, 2009), which, including need to belong among the primary motivations, makes predictions consistent with the need-to-belong theory (Baumeister and Leary, 1995). According to Williams’ model, individuals detect the occurrence of an ostracism episode very rapidly, their immediate reactions being pain, negative affect, and need frustration (the reflexive stage of reaction to ostracism). The function of this stage is to evaluate the importance of the ostracism event and to plan coping strategies. In the workplace, these unpleasant reactions can explain the negative correlations of ostracism with job satisfaction and organizational identification (Ferris et al., 2008). Frequent episodes of exclusion can lead to chronic stress and emotional exhaustion.

In the subsequent reflective stage, excluded individuals try to fortify the frustrated needs. To reinforce the need to control, they may use aggression toward excluding people or naive others. In the workplace, aggression can promote deviant behaviors toward the organization or other employees (see Ferris et al., 2008). In contrast, to reinforce the needs to belong and self-esteem, excluded individuals may use compliance or positive behaviors, such as altruism or working harder for the team (Williams, 2009). Actually, in the workplace, ostracism is negatively related to OCBs and performance, probably because excluded workers do not have enough energies to engage in constructive tasks or have turnover intentions. Persistent experiences of ostracism over time may deplete the resources needed to fortify the frustrated needs, thus leading to depression and alienation (the resignation stage).

Given the importance of ostracism, Ferris et al. (2008) developed the workplace ostracism scale (WOS), a 10-item unidimensional tool, not affected by method factors, showing convergent, discriminant, and criterion validity. In the present study, we aim to test the validity of the WOS in the Italian social context. We will assess its dimensionality, reliability, and invariance across genders and ages. In addition, we will test its nomological validity, that is, whether ostracism, as measured by the WOS, is embedded in a network of constructs that are related to ostracism on a theoretical or empirical basis (for the concept of nomological validity, see Bagozzi, 1981). To identify the constructs related to workplace ostracism (WO), we examined recent meta-analyses and systematic reviews.

### 1.1 Antecedents and outcomes of workplace ostracism

In the first meta-analysis on workplace ostracism, Howard et al. (2020) reviewed 93 studies concerning the antecedents and outcomes of this variable. They found that precursors of ostracism are four dimensions of the Big Five: ostracism is negatively related to extraversion, conscientiousness, and agreeableness; it is positively related to neuroticism. Among demographics, felt ostracism shows a small, but reliable, relationship with gender and working full- versus part-time: men and part-time employees report more ostracism than women and full-time employees. However, the strongest predictor of ostracism is leadership: feelings of exclusion are associated with supervisor’s ostracism behaviors and abusive supervision, which is characterized by mistreatment and hostile attitudes.

Regarding consequences, workplace ostracism is negatively related to performance and helping; it is, in contrast, positively related to deviant, counterproductive behaviors, such as knowledge hiding (see Zhao et al., 2013, 2016; Fatima, 2016) and voice, namely the allocation of less effort in performing one’s work. For well-being, ostracism is positively related to depression, job burnout^1^ (see Sulea et al., 2012), and job tension, whereas it is negatively related to life satisfaction and positive affect. As expected, feelings of exclusion are negatively related to job perceptions, for instance: job satisfaction, occupational commitment (see O’Reilly et al., 2015), and work engagement (Leung et al., 2011), the latter being a positive work-related mental state, characterized by vigor, dedication, and absorption in one’s work (Schaufeli et al., 2002). For exit behaviors, ostracism shows a large, positive relationship with turnover intentions and a small, significant relationship with turnover. Notably, Howard et al. (2020) discovered that performance, helping, and deviant behaviors may be both outcomes and antecedents of workplace ostracism.

Li et al.’s (2021) meta-analysis focused on the consequences of workplace ostracism. Their findings, based on 95 independent samples, replicate those by Howard et al. (2020). Li and colleagues discovered that ostracism is negatively related to job performance and OCBs; it is positively related to turnover intentions and counterproductive behaviors. As to job perceptions and well-being, ostracism is negatively related to job satisfaction, whereas it is positively related to burnout. As expected, individualism–collectivism (Hofstede, 2001) is a significant moderator; for instance, the negative relationship between ostracism and prosocial behaviors (OCBs) directed to individual employees is stronger in individualistic countries, whereas the negative relationship between ostracism and organizational identification is stronger in collectivist countries.

The meta-analytic review by Bedi (2021), based on 100 independent samples, supported the above findings. Bedi discovered that antecedents of ostracism are personality characteristics; WO is, for instance, positively correlated with negative affectivity and neuroticism, whereas it is negatively correlated with agreeableness, extraversion, conscientiousness, and social skills. For the outcomes, ostracism is associated with health conditions: it is positively related to emotional exhaustion and negatively related to psychological capital (e.g., optimism and resilience). Perceived ostracism is, in addition, negatively linked to organizational identification and commitment. Finally, WO is negatively associated with performance and prosocial behaviors.

Kaushal et al.’s (2021) literature review was conducted using a combination of methods: content analysis, network analysis, and bibliometrics—a field study using statistical and mathematical methods to analyze books, articles, and other publications (Donohue, 1973); bibliometrics is an appropriate tool for reviewing the development of a research field (Castriotta et al., 2019). In the selected articles (*N* = 144), the authors identified three clusters of themes. The first focused on the motivational consequences of feeling excluded. The main theoretical frameworks were the temporal model of need threat (Williams, 2009) and the need-to-belong theory (Baumeister and Leary, 1995). The second cluster was grounded in social exchange theories (Gouldner, 1960; Blau, 1964). Findings showed that ostracism may originate from perceiving the excluded employee as a weak partner in the exchange (e.g., Scott et al., 2013); ostracized employees, in turn, may react to exclusion by reducing citizenship behaviors (for moderators of the latter effect, see Balliet and Ferris, 2013). The third cluster concerned method problems, namely, the development of the WOS by Ferris et al. (2008) and the extent to which method biases (Podsakoff et al., 2003) may affect the research on ostracism.

Recently, Asmita et al. (2024) performed a literature review and bibliometric analysis, considering 134 articles published between 2000 and 2023. This review expands on the work by Kaushal et al. (2021) by including papers published after 2020. Regarding WO’s consequences, Asmita et al. confirmed the findings of previous analyses. Feeling ostracized has unfavorable effects on health: it increases tension, psychological distress, emotional exhaustion, and depression. WO has also unfavorable effects on job attitudes and perceptions: it reduces job satisfaction, organizational commitment, and commitment to managers. In addition, it negatively impacts performance, weakening creative thinking, innovative work, cooperation, and proactivity; at the same time, it increases counterproductive behaviors and social loafing. WO is negatively related to citizenship behaviors.

Asmita et al. (2024) examined the mediation processes through which workplace ostracism affects employees. They found that the main mediators are: basic need frustration, stress, emotional exhaustion, and lower work engagement.

The previously mentioned reviews consistently show that feeling ostracized is positively related to exhaustion (burnout) and negatively related to work engagement, life and job satisfaction; it is negatively associated with citizenship behaviors and job performance. Supporting Williams’ (2009) model, Kaushal et al.’s (2021) and Asmita et al.’s (2024) reviews clarified that basic need frustration is an important variable associated with ostracism. Feeling ostracized is thus connected to central concepts in the job demands-resources (JD-R) theory (Bakker and Demerouti, 2017; Bakker et al., 2023); therefore, we will use this theory to evaluate the nomological validity of the Italian version of the WOS.

### 1.2 The job demands-resources theory

The JD-R theory (Bakker and Demerouti, 2017; Bakker et al., 2023) is an influential explanation of work-related well-being and organizational performance. According to the JD-R theory, all job characteristics can be modeled using two distinctive categories, namely job demands and job resources. Job demands, such as work overload and interpersonal conflicts, can be defined as the physical, organizational, social, or psychological facets of the job that require an effort and are, therefore, associated with physiological and/or psychological costs (Bakker et al., 2023). Job demands, draining employees’ resources, may lead to burnout and health issues (health impairment process).

Job resources, such as social support and performance feedback, are the physical, organizational, social, or psychological facets of the job that have motivating potential, are functional in achieving one’s goals, regulate the effects of demands, and foster employees’ personal growth (Bakker et al., 2023). Job resources, having motivational potential, may lead to work engagement—vigor, dedication, and absorption in one’s work (motivational process).

Consistent evidence supports the two processes: resources are related to higher work engagement and lower burnout, whereas demands are related to higher burnout and lower engagement (see Alarcon, 2011; for meta-analyses, see Christian et al., 2011; Nahrgang et al., 2011; Lesener et al., 2020). The negative link between demands and work engagement, not suggested by the theory, has been found in several studies and meta-analyses (see, e.g., Crawford et al., 2010; Nahrgang et al., 2011; Capozza et al., 2023).

Concerning behaviors, it has been discovered that work engagement mediates the positive relationship between resources and task and contextual performance (Christian et al., 2011). The former refers to the tasks required by a job; the latter concerns behaviors that, although not formally required, are useful in shaping the social climate of an organization (an example is organizational citizenship behavior; for this distinction, see Borman and Motowidlo, 1997). For other types of behaviors, Bakker et al. (2023) revealed that burnout mediates the association between demands and absence duration. Swider and Zimmerman (2010) found a positive link between emotional exhaustion and turnover. In testing a meta-analytic path model, Nahrgang et al. (2011) discovered that resources, such as safety climate, were negatively related to adverse events (errors and near misses) through the mediation of higher work engagement and lower burnout; conversely, demands (e.g., risks and hazards) were positively linked to adverse events only through the mediation of lower work engagement.

Thus, resources are related to citizenship behaviors, improved performance, and fewer errors in working through the mediation of higher engagement and lower burnout. Demands are related to decreased performance and dysfunctional behaviors through the mediation of higher burnout or weaker engagement.

The motivational and health impairment processes can be explained by the satisfaction of basic psychological needs, which is facilitated by resources (see Proposition 2 in Bakker et al., 2023) and reduced by demands. Satisfaction of basic needs is a central concept in the self-determination theory (SDT; Deci and Ryan, 2000; Ryan and Deci, 2000, 2017), a prominent explanation of motivation and well-being. According to the SDT, need satisfaction is essential for humans to realize their potential, grow and flourish, and prevent maladaptive functioning. Three basic needs are postulated: autonomy, relatedness, and competence (Deci and Ryan, 2000). The need for autonomy is satisfied when individuals feel that what they are doing is freely chosen (Deci and Ryan, 2000). The need for relatedness is the human striving for close and intimate relationships (Baumeister and Leary, 1995). Regarding the need for competence, individuals wish to feel capable of mastering their environment, achieving the desired outcomes, and managing the challenges that arise (White, 1959). Of these needs, relatedness and competence correspond to two of the needs conceptualized in Williams’ (2009) model of ostracism: belongingness and control, respectively.

The intervening role of need satisfaction was shown by Van den Broeck et al. (2008). In a correlational study, they discovered that the satisfaction of basic needs mediated the relationship between resources and work engagement, whereas need frustration mediated the relationship between demands and burnout (emotional exhaustion). Furthermore, need satisfaction explained the negative relationship between resources and exhaustion and need frustration explained the negative relationship between demands and work engagement. These findings were fully replicated in an experimental investigation performed by Capozza et al. (2023).

Indirect evidence for the mediation role of basic needs derives from Van den Broeck et al.’s (2016) meta-analysis. These authors found that job demands were related to the frustration of all three needs, whereas job resources were related to the satisfaction of the three needs. Need satisfaction, in turn, was positively correlated with work engagement and negatively correlated with burnout.

In this paper, we conceptualize work ostracism as a job demand. Consistent with Bakker and colleagues’ definition of demand (Bakker and Demerouti, 2017; Bakker et al., 2023), WO can be defined as a socio-relational aspect of the job that requires sustained emotional and cognitive effort. Furthermore, WO shows the same associations with other concepts as job demands: it is negatively related to basic need satisfaction (see, e.g., the review by Kaushal et al., 2021), positively related to burnout and negatively to work engagement (see, e.g., Asmita et al., 2024). WO hinders job performance and helping behaviors; like job demands, ostracism facilitates counterproductive behaviors (see, e.g., Asmita et al., 2024).

Therefore, to evaluate the nomological validity of the Italian version of the WOS (Ferris et al., 2008), we embedded ostracism into the job demands-resources theory. The Italian version of the WOS has nomological validity if it allows us to detect the relationships between constructs that are predicted by the JD-R theory or, although not predicted, are reliably observed in the theory validation process (e.g., the negative relationship between job demands and work engagement).

In this study, we thus propose the following hypotheses.

*Hypothesis 1.* As for the original scale by Ferris et al. (2008), the Italian WOS will have a unidimensional structure and will be free from social desirability issues. Moreover, factor structure will be invariant across genders and ages.

*Hypothesis 2.* Ostracism, as measured by the Italian WOS, will be included in the following network of relationships.

*Hypothesis 2a.* It will be directly and negatively related to basic need satisfaction.

*Hypothesis 2b.* It will be indirectly and negatively related to employees’ well-being. Need frustration will have an intermediate role in this relationship.

*Hypothesis 2c.* Ostracism will be indirectly and negatively related to job performance and citizenship behaviors. Need frustration and low well-being will have an intermediate role in these relationships.

Validation of Hypothesis 2 will support the nomological validity of the Italian WOS. We do not formulate this hypothesis in terms of mediation because, in processing data, we will use network analysis instead of the standard methods for testing mediation—regression, bootstrapping, and confidence intervals.

## 2 Overview of the study

In this study, we examined a sample of Italian employees working in different organizations and several Italian regions. They completed an online questionnaire that included the Italian version of the ostracism scale (Ferris et al., 2008) and measures of job characteristics. Two demands and two resources were included. Demands were work overload and role ambiguity (i.e., poorly defined activities; see Brauchli et al., 2015, for a study using workers from different sectors). Resources were autonomy and social support from colleagues (see Van den Broeck et al., 2008, for a survey conducted in 17 different organizations). Therefore, job aspects were chosen that reliably affect well-being when, as in our case, employees belonging to several work sectors are examined (see also Van den Broeck et al., 2017, and Colledani et al., 2024, for the Italian work context). The four job aspects were introduced in the study to control their effects and, thus, identify ostracism unique associations with basic needs, well-being, and behaviors (Hypothesis 2).

In analyzing data, we averaged the three needs, aiming to obtain a single measure and, thus, a more simplified representation of the network of relationships. Notably, the procedure of averaging needs is not unusual in ostracism research (see Bernstein et al., 2010, 2021; Sacco et al., 2014).

In measuring well-being, we may have used both an indicator of health impairment (burnout) and an indicator of vigor and dedication to work (work engagement). We only used work engagement for two reasons: 1. to simplify the representation of the network; 2. because it has been found that burnout (exhaustion) may be unrelated to performance and helping behaviors (see Capozza et al., 2023). Burnout is generally related to performance when the latter is measured using observers’ ratings (Swider and Zimmerman, 2010) or objective indicators of productivity (see the review by Patel et al., 2018). In the present study, we only used self-report measures.

To investigate the dimensionality of the Italian WOS, both exploratory factor analysis (EFA) and confirmatory factor analysis (CFA) were used. Two samples were obtained by randomly dividing participants into two groups of a similar size. The first was used to run parallel analysis (PA) and exploratory factor analysis: parallel analysis allowed us to establish the number of factors; EFA was run asking for the number of factors suggested by PA. These exploratory analyses were pertinent given that our study was the first Italian validation of the WOS. Confirmatory factor analysis (second sample) was modeled based on EFA findings. These factor analyses, conducted with different methods and samples, increase confidence in the dimensionality of the scale and provide evidence concerning its stability.

The complete data set was used to assess the measurement invariance of the WOS across genders and age groups (employees up to, and over, 40 years old). Configural invariance (same pattern of fixed and free parameters), metric invariance (equality of factor loadings), scalar invariance (equality of both factor loadings and item intercepts), and strict invariance (equality of factor loadings, intercepts, and residual variances) were tested. Detecting measurement invariance is a crucial step in the validation of a scale allowing us to know whether the new instrument has the same functioning across different groups. Measurement invariance is the premise for meaningful comparisons across groups (Vandenberg and Lance, 2000; Colledani et al., 2021, 2024; Anselmi et al., 2022).

Network analysis was applied to establish the scale nomological validity. Network analysis is a valid approach for testing this type of validity because it allows for an exploration of the relationships between constructs in a theoretical framework. In the next paragraph, some basic concepts of this method will be reported.

## 3 Network analysis concepts

In the past two decades, network analysis has become an important conceptual and analytical approach in psychological research. Although this approach is longstanding, given that it was applied in causal attribution studies (Kelly, 1983) and social network analysis, its broader potential was highlighted almost 20 years ago by van der Maas et al. (2006) when explaining general intelligence. The basic concept in network analysis is that psychological phenomena are complex systems in which the constituent parts influence each other. Indeed, the relationship between psychological variables is often bidirectional and a change in a variable can trigger a change in both its antecedents and outcomes. In the JD-R theory, for instance, a distal precursor of work engagement is job crafting, namely employees’ initiative to change job demands and resources to better align them to their personal needs and abilities (Tims and Bakker, 2010). Engaged employees, in turn, being intrinsically motivated to stay engaged, use job crafting to optimize their work conditions and performance. Thus, work engagement is both a consequence and a cause of job crafting; it is embedded in a network containing various constructs and mutual relationships (in this work, we did not include job crafting wishing to simplify the conceptual and statistical analyses).

Considering their features, networks are structures comprising concepts, represented by nodes, and relationships between concepts, represented by edges (see the Results section: Nomological validity). Edges may be directed or undirected; in the latter case, nodes have a connecting line with no arrowheads, indicating a mutual relationship. Generally, undirected networks are used (Hevey, 2018). Edges may be positive or negative; the sign of the relationship may be graphically represented using different lines: positive relationships can be drawn as continuous lines and negative relationships as dotted lines. The strength of the edge is measured as the partial correlation between the two nodes, controlling for the other nodes in the network. An edge, therefore, indicates a unique association between two variables, not affected by the other variables of the system. The strength of an edge is graphically represented by varying its thickness and density: thicker and denser lines indicate stronger associations.

Some variables in the network have more connections than others, that is, some nodes are more central than others. Several centrality indices have been proposed; the most commonly used are strength, closeness, and betweenness. The first index measures the strength, in absolute terms, of the direct connections of a node with the other nodes; higher values signify that a node has numerous strong direct relationships with the other nodes. The closeness index quantifies how strongly a node is linked directly or indirectly to all the other nodes in the network. A variable with high closeness will be quickly affected by changes in other parts of the system; its changes, in turn, will quickly affect the other parts (Borgatti, 2005). Finally, betweenness measures how often a node lies on the shortest path between two other nodes. A higher score indicates that a node plays a key role in connecting the other nodes.

Regarding this intermediate role, compared to the standard mediation models, that rely on linear recursive paths, network analysis has the potential to explore both unidirectional and bidirectional mediation effects. This is particularly useful when theories including bidirectional relationships are tested. Using network analysis in the context of the JD-R theory, we can, for instance, explore the idea that work engagement plays a mediation role both in the path from need satisfaction to higher performance and in the path from higher performance to need satisfaction. With the standard mediation model, two alternative models should be run. In addition, network analysis provides a measure of the degree to which a concept functions as a mediator (i.e., is central) in a theoretical system (the betweenness index).

For centrality, we hypothesize that a node with high centrality will be need satisfaction (Hypothesis 3). In fact, it should be directly and strongly related to job demands, job resources, and work engagement, playing—in the JD-R theory—a mediation role between work characteristics and well-being (i.e., it should have high strength centrality). In addition, as a mediator in the theory, it should be located in the shortest path between numerous pairs of variables, namely, between job characteristics and work engagement; between job characteristics and outcomes (performance and OCBs) (i.e., it should have a high betweenness score). Finally, it should be close to numerous variables in the net, the greatest distance probably regarding the two outcomes (i.e., it should have high closeness).

## 4 Method

### 4.1 Participants

A total of 653 participants took part in the study. The sample included 336 women (51.50%) and 317 men (48.50%), aged between 18 and 65 (*M* = 38.19, *SD* = 13.35) years. Most respondents had completed high school (55.74%), 10.57% had basic education (primary or junior high school), and 33.69% had a university degree or higher qualifications. Regarding the professional level, participants had to choose one of four alternatives: “white-collar worker,” “blue-collar worker,” “manager,” and “other position.” The majority (43.03%) chose white-collar worker, 25.27% blue-collar worker, and 5.05% chose manager. The remaining participants (26.65%) answered that they belonged to specific sectors, such as education, healthcare, and trade. For length of service, most participants (58.81%) reported a seniority of 10 years or less, 17.61% a seniority ranging from 11 to 20 years; the remaining participants (23.58%) had been working in their organization for over 20 years.

All respondents were Italian; they were recruited from different Italian regions and different organizations through an online survey. A snowball sampling procedure was used. Before accessing the questionnaire, participants were required to provide electronic informed consent; they were informed about the goals of the study, the duration of the task, and their right to withdraw their participation. The study was performed adhering to ethical principles, as outlined in the Helsinki Declaration for research involving human subjects; it was approved by the local Ethical Committee for Psychological Research.

### 4.2 Measures

Participants were presented with a questionnaire including scales and questions regarding demographic characteristics, such as gender, age, length of service, and professional level. The following measures were used.

#### 4.2.1 Workplace ostracism

The workplace ostracism scale (WOS; Ferris et al., 2008) was applied to assess respondents’ perceptions of exclusion, neglect, and social isolation in the work environment. The scale consists of 10 items (e.g., “How often did others ignore you at work?” “How often did others avoid you at work?” “How often did others shut you out of the conversation at work?” “How often did others refuse to talk to you at work?”). The stem sentence was: “Please, indicate how often, in the work environment, you have experienced the following situations.” The 7-point response scale was: 1 = *never*, 2 = *once in a while*, 3 = *sometimes*, 4 = *fairly often*, 5 = *often*, 6 = *constantly*, 7 = *always*. Items were translated from English into Italian by two Italian investigators, and then back-translated by a native English speaker, to ensure linguistic equivalence. The Italian WOS’s reliability was high (alpha = 0.86).

#### 4.2.2 Job demands

For job demands and job resources, we used items drawn from different sources (e.g., Van den Broeck et al., 2008, 2017; Fernet et al., 2013; Kaiser et al., 2020). We considered two demands: work overload and role ambiguity. Work overload was measured with four items, for instance: “At work, what I have to do is often complex,” “At work, I generally have to work under tight time deadlines” (alpha = 0.62, after the deletion of one item). Four items were used for role ambiguity, for instance: “At work, the activities I have to perform are not clearly defined,” “The roles I have to play and the rules I have to follow are not clear” (alpha = 0.74). For demands and resources, responses were coded on a 7-point scale ranging from 1 (*definitely false*) to 7 (*definitively true*) with 4 denoting *neither true nor false*. Higher numbers indicate higher levels of perceived demands.

#### 4.2.3 Job resources

Job resources were social support from colleagues and autonomy. Social support was measured using three items, for instance: “In my work environment, there is at least one employee I can ask for advice” (alpha = 0.79). The autonomy scale also included three items, for instance: “At work, I have some freedom in the completion of my tasks” (alpha = 0.83). Higher numbers indicate higher levels of perceived resources.

#### 4.2.4 Basic need satisfaction

This construct was measured using the Italian version (Colledani et al., 2018) of the Work-related Basic Need Satisfaction (W-BNS) scale, developed by Van den Broeck et al. (2010). To shorten the scale, for each need, we selected three of the six representative items: those with higher loading on the respective factor (see Colledani et al., 2018). Sample items are: “I feel free to do my job the way I think it could best be done” (need for autonomy; alpha = 0.67); “At work, I can talk with people about things that really matter to me” (need for relatedness; alpha = 0.59); “I feel competent at my job” (need for competence; alpha = 0.81). The reliability of the whole scale was alpha = 0.80. The 7-point response scale was anchored by *completely disagree* and *completely agree*. Higher scores express higher satisfaction of basic needs.

#### 4.2.5 Work engagement

This variable was assessed through the Italian version (Balducci et al., 2010) of the shortened Utrecht Work Engagement Scale (UWES-9; Schaufeli et al., 2006), which measures the three facets of the construct: vigor, dedication, and absorption (nine items). Examples of items are: “At my job, I feel strong and vigorous,” “My job inspires me,” “I feel happy when I am working intensely” (alpha = 0.93). Answers were given on a 7-point scale anchored by *never* (1) and *daily* (7) (2 = *rarely/a few times a year or less*, 3 = *occasionally/once a month or less*, 4 = *regularly/a few times a month*, 5 = *frequently/once a week*, 6 = *very frequently/a few times a week*). Higher scores denote higher work engagement.

#### 4.2.6 Organizational citizenship behavior

This construct was measured using items partly taken from Pond et al. (1997). Three assess altruism, namely prosocial behaviors directed to single employees, such as “At work, I help others who have a heavy workload.” Three measure compliance, namely prosocial behaviors directed to the entire organization, such as “At work, I make suggestions on how to improve services.” Four items, derived from Choi (2007), measure citizenship actions directed to organizational change, for instance: “I often suggest work improvement ideas to others,” “I often suggest changes of unproductive rules or policies.” A 7-point scale was used ranging from 1 (*definitely false*) to 7 (*definitely true*) with 4 denoting *neither true nor false*. Higher scores indicate higher levels of citizenship behavior. The reliability of the whole scale was high (alpha = 0.84).

#### 4.2.7 Job performance

The self-reported performance was measured using a scale elaborated by Abramis (1994). Examples of the four items are: “In the last seven days you worked, how well were you handling the responsibilities and daily demands of your work?” “How well were you performing without mistakes?” (alpha = 0.78). The anchors of the 5-point response scale were *very poorly* (1) and *very well* (5). Higher numbers indicate self-reported higher performance.

#### 4.2.8 Desirable responding

To assess social desirability, we applied the impression management subscale of the Balanced Inventory of Desirable Responding (Paulhus, 1991). This scale measures the inclination to provide positively inflated self-descriptions. We used the abbreviated Italian version elaborated by Bobbio and Manganelli (2011), which includes eight items, for instance: “I always obey laws, even if I am unlikely to get caught,” “I have taken sick leave from work or school even though I wasn’t really sick” (reverse coded). The 6-point scale ranged from 1 (*strongly disagree*) to 6 (*strongly agree*). The alpha coefficient was 0.68.

## 5 Results

### 5.1 Factor structure of the WOS

As previously mentioned, participants were randomly divided into two samples. Data from one sample (*n* = 327; 52.00% women; *M*age = 38.50, *SD* = 13.44; 10-item alpha = 0.86) were analyzed using parallel analysis and exploratory factor analysis (Kaiser-Meyer-Olkin = 0.91; Bartlett’s test of sphericity: χ^2^ = 1347.57, *df* = 45, *p* < 0.001). Mplus (Muthén and Muthén, 1998–2015) was applied and the robust maximum likelihood (MLR; Yuan and Bentler, 2000) was used as estimator (Geomin rotation).

**Figure 1:**
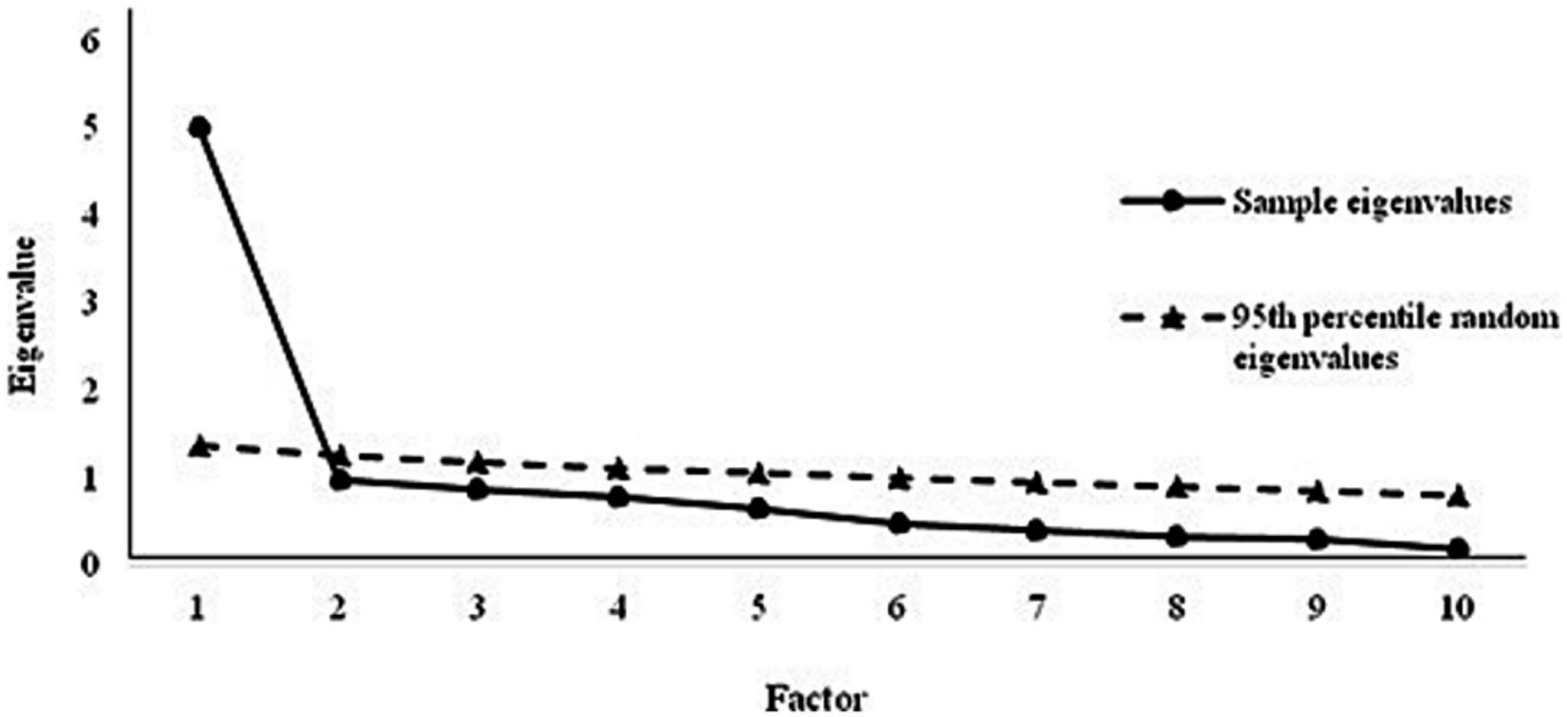
Scree plot of real data eigenvalues and 95th percentile of eigenvalues from random data (*n* = 327).

Parallel analysis highlighted that the 10 items of the WOS only measured one factor: in fact, only the first eigenvalue (sample eigenvalue) was higher than the parallel eigenvalue (Figure 1) obtained from random data sets (1,000 resamples). For each factor, the 95th percentile of the randomly generated eigenvalues was used, rather than the mean, because it leads to a more conservative estimate of the random data distribution. EFA showed that factor loadings of the 10 items in the unidimensional structure were all significant (*p* < 0.001) (Table 1).

**Table 1:** Unifactorial structure of the Italian version of the WOS: EFA (*n* = 327) and CFA (*n* = 326) findings.

Items	EFA loading	CFA loading
Item 1	0.65	0.58
Item 2	0.26	0.40
Item 3	0.48	0.55
Item 4	0.59	0.41
Item 5	0.86	0.81
Item 6	0.89	0.82
Item 7	0.78	0.80
Item 8	0.69	0.70
Item 9	0.77	0.72
Item 10	0.53	0.64

All loadings are significant at *p* < 0.001; item numbers correspond to those of the scale developed by Ferris and colleagues (see the Appendix in Ferris et al., 2008); WOS, workplace ostracism scale; EFA, exploratory factor analysis; CFA, confirmatory factor analysis.

Data from the second sample (*n* = 326; 50.92% women; *M*age = 37.88, *SD* = 13.26; 10-item alpha = 0.87) were analyzed using CFA (Mplus; MLR as estimator; Kaiser-Meyer-Olkin measure = 0.90; Bartlett’s test of sphericity: χ^2^ = 1549.84, *df* = 45, *p* < 0.001). To evaluate the one-factor solution, several goodness-of-fit indices were applied: χ^2^; χ^2^/*df* ratio (Kline, 2010); standardized root mean square residual (SRMR; Bentler, 1995); root mean square error of approximation (RMSEA; Browne and Cudeck, 1993); comparative fit index (CFI; Bentler, 1990). The adequacy of a model is supported by a nonsignificant χ^2^, a CFI close to 0.95, a SRMR value less than 0.08, and a RMSEA less than 0.06 (see Hu and Bentler, 1999; Marsh et al., 2004). For the χ^2^/*df* ratio, it should be less than 3 (Kline, 2010). The one-factor solution explained the data very well. In fact, fit indices satisfied the respective criterion: CFI = 0.966, SRMR = 0.038, RMSEA = 0.043. Chi-square—χ^2^ (35) = 56.12, *p* = 0.013—was significant, but the χ^2^/*df* ratio was less than 3. Factor loadings, all reliable, ranged from 0.40 to 0.82 (Table 1). All findings converge in supporting the unifactorial structure of the Italian WOS.

To establish the reliability of the scale and its independence from social desirability issues, data from the entire sample (*N* = 653) were considered. Reliability was high both when using alpha (0.86) and when using composite reliability (CR = 0.88). The independence of the WOS from desirable responding was shown by its low correlation with the impression management scale (*r* = 0.21, *p* < 0.001). This relation indicates that social desirability only absorbed 4% of the responses to the WOS. Thus, findings supported Hypothesis 1: the Italian WOS proves to be a reliable, unidimensional tool, rather independent from social desirability issues (the Italian version of the scale and all data from this study are available upon request from the corresponding author).

### 5.2 Measurement invariance

The measurement invariance of the WOS was assessed considering genders and two age groups (employees up to, and over, 40 years). Configural, metric, scalar, and strict models were run using the data from the entire sample (*N* = 653). To assess the equivalence of nested models, changes in S-B χ^2^ (Satorra-Bentler scaled chi-square; Satorra and Bentler, 1994), CFI, RMSEA, and SRMR were evaluated: ΔS-B χ^2^, ΔCFI, ΔRMSEA, ΔSRMR (for changes in CFI, RMSEA, and SRMR, see Cheung and Rensvold, 2002; Chen, 2007). Invariance is supported by nonsignificant ΔS-B χ^2^ values and ΔCFI values ≤ |0.010|, paired with ΔRMSEAs ≤ |0.015|. For ΔSRMRs, they should be ≤ |0.030|, for metric invariance, ≤ |0.015|, for scalar invariance, and ≤ |0.010|, for strict invariance.

Following these rules of thumb, configural invariance (same pattern of fixed and free parameters) and metric invariance (equality of factor loadings) were observed across genders and age groups (see Table 2). Conversely, only partial scalar invariance (equality of both factor loadings and intercepts) and strict invariance (equality of factor loadings, intercepts, and residual variances) were observed for both comparisons (Table 2). Specifically, for genders, all intercepts were invariant except for item 7, which had a lower intercept among females. For age groups, all intercepts were invariant except for item 2, which had a lower intercept among employees aged over 40. Concerning strict invariance, differences in residual variances were observed for item 8 across both genders and age groups, suggesting that, while this item equally measured the common factor, it was differently affected by disturbance factors across the compared groups.^2^ Overall, the Italian WOS largely maintained the same meaning and functioning across the two comparisons.

**Table 2:** Fit indices of invariance testing.

Invariance	χ^2^	*df*	*p* <	RMSEA	CFI	SRMR	∆S-B χ^2^	∆*df*	*p*-value =	∆CFI	∆RMSEA	∆SRMR
Gender (M = 317; *F* = 336)												
Configural	131.134	70	0.001	0.052	0.956	0.040						
Metric	141.481	79	0.001	0.049	0.955	0.065	12.884	9	0.168	0.001	0.003	−0.025
Scalar	160.816	88	0.001	0.050	0.947	0.064	21.943	9	0.009	0.008	−0.001	0.001
Scalar (7)	151.443	87	0.001	0.048	0.953	0.063	6.642	8	0.576	0.002	0.001	0.002
Strict	142.225	96	0.002	0.038	0.967	0.069	5.390	9	0.799	−0.014	0.010	−0.006
Strict (8)	144.750	95	0.001	0.040	0.963	0.066	4.360	8	0.823	−0.010	0.008	−0.003
Age (up 40 years old = 332; over 40 years old = 321)												
Configural	129.420	70	0.001	0.051	0.956	0.039						
Metric	136.878	79	0.001	0.047	0.957	0.057	11.007	9	0.275	−0.001	0.004	−0.018
Scalar	155.328	88	0.001	0.048	0.950	0.062	20.649	9	0.014	0.007	−0.001	−0.005
Scalar (2)	148.840	87	0.001	0.047	0.954	0.059	10.467	8	0.234	0.003	0.000	−0.002
Strict	172.201	96	<0.001	0.049	0.944	0.076	18.997	9	0.025	0.010	−0.002	−0.017
Strict (8)	157.126	95	<0.001	0.045	0.954	0.071	11.258	8	0.188	0.000	0.002	−0.012

Multigroup confirmatory factor analysis was used to compare subgroups of participants. RMSEA, root mean square error of approximation; CFI, comparative fit index; SRMR, standardized root-mean-square residual; ∆S-B χ^2^, Satorra-Bentler scaled chi-square difference test; ∆RMSEA, test of change in RMSEA; ΔCFI, test of change in CFI; ∆SRMR, test of change in SRMR. For scalar and strict invariance, the numbers in parentheses indicate noninvariant intercepts or residual variances. In all models, the χ^2^/*df* ratio was lower than 2.

### 5.3 Nomological validity of the WOS

To evaluate the WOS nomological validity, we applied network analysis (whole sample data). The extended Bayesian information criterion (EBIC) and the graphical LASSO (least absolute shrinkage and selection operator) were used with the *γ* hyperparameter set to 0.50 (Chen and Chen, 2008; Friedman et al., 2008; Foygel and Drton, 2010). This procedure shrinks the number of edges when evaluating a network model, which means that small edges are estimated to be exactly zero. This regularization process leads to sparse networks in which likely spurious connections are removed. Analyses were performed using the R (R Core Team, 2021) package bootnet (Epskamp et al., 2018).

**Figure 2:**
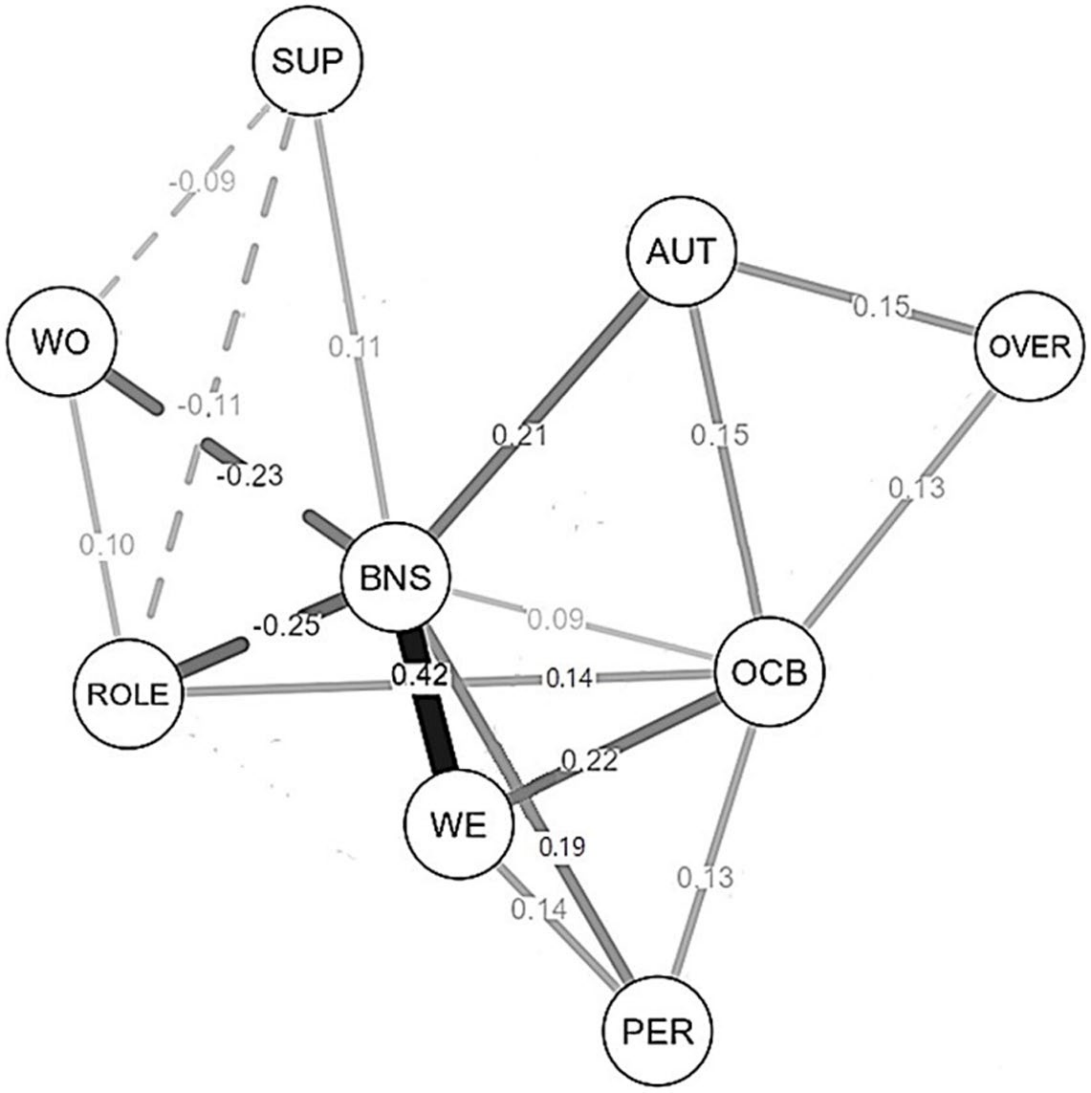
The network of relationships between core concepts in the job demands-resources theory, including workplace ostracism (*N* = 653). In the network, lines represent edges (regularized partial correlations) between concepts (nodes). Thicker and denser lines represent stronger—positive (continuous line) or negative (dotted line)—connections. For the sake of simplicity, only significant (*p* < 0.05) correlations are displayed, namely correlations ≥ |0.09|. WO, workplace ostracism; SUP, social support from colleagues; ROLE, role ambiguity; AUT, autonomy; OVER, work overload; BNS, basic need satisfaction; WE, work engagement; OCB, organizational citizenship behavior; PER, self-reported performance.

The network of concept relationships is shown in Figure 2. Nodes correspond to concepts and lines to edges; thicker and denser lines represent stronger partial correlations (positive or negative; Table 3). To simplify the graph, only significant coefficients are displayed in Figure 2 (partial *r* ≥ 0.09, *p* < 0.05).

**Table 3:** Descriptive statistics and regularized partial correlations between concepts (*N* = 653).

Concept	*M*	*SD*	1	2	3	4	5	6	7	8	9
1. Workplace ostracism	1.60	0.66	–								
2. Role ambiguity	2.80	1.34	*0.10*	–							
3. Work overload	4.61	1.33	0.00	0.00	–						
4. Autonomy	5.31	1.41	−0.06	0.00	*0.15*	–					
5. Social support	5.93	1.25	*−0.09*	*−0.11*	0.00	−0.07	–				
6. Basic need satisfaction	5.36	0.89	*−0.23*	*−0.25*	0.00	*0.21*	*0.11*	–			
7. Work engagement	4.28	1.20	0.00	−0.06	0.03	0.06	0.00	*0.42*	–		
8. OCB	5.33	0.86	0.00	*0.14*	*0.13*	*0.15*	0.00	*0.09*	*0.22*	–	
9. Performance	3.96	0.60	0.00	−0.04	0.02	0.00	0.00	*0.19*	*0.14*	*0.13*	–

*M*, mean; *SD*, standard deviation; OCB, organizational citizenship behavior. Significant correlations (*p* < 0.05) are in italics.

Visual inspection of the network shows that workplace ostracism, measured by the Italian WOS, was directly connected to the frustration of basic needs (Hypothesis 2a). Furthermore, basic need frustration was the bridge connecting ostracism to lower well-being (work engagement) (Hypothesis 2b). In addition, both need frustration and lower engagement linked ostracism to the reported behaviors—performance and OCB (Hypothesis 2c). Thus, the Italian WOS shows nomological validity: it allowed us to replicate relationships that are included in the JD-R theory (e.g., the positive relationship between basic need satisfaction and work engagement; Bakker et al., 2023) or relationships that are consistently observed in studies aimed to validate the theory (e.g., the negative—direct or indirect—link between job demands and work engagement; see, e.g., Crawford et al., 2010; Capozza et al., 2023).

Note the unexpected direct connections between the node representing need satisfaction and the nodes representing OCB and performance (Figure 2). Need satisfaction has a central position in the network. It exhibits: 1. the highest strength centrality index (Table 4), having the highest number of direct connections with the other concepts, four of which being of medium size (from 0.20 to 0.50; see Ferguson, 2016); 2. the highest closeness index, showing the shortest distance from all the other concepts; 3. the highest betweenness index, being the bridge connecting numerous pairs of concepts. Thus, data supported Hypothesis 3: satisfaction of basic needs is central in explaining well-being (work engagement) and its outcomes, a finding coherent with both the self-determination theory (e.g., Ryan and Deci, 2017) and the JD-R theory (Bakker et al., 2023).

**Table 4:** Centrality indices from network analysis (*N* = 653).

Concept	Strength	Closeness	Betweenness
Workplace ostracism	0.32	0.63	0.00
Role ambiguity	0.48	0.72	0.07
Work overload	0.23	0.47	0.00
Autonomy	0.46	0.72	0.20
Social support	0.25	0.46	0.00
Basic need satisfaction	1.00	1.00	1.00
Work engagement	0.62	0.83	0.13
OCB	0.58	0.71	0.20
Performance	0.35	0.62	0.00

OCB, organizational citizenship behavior.

Regarding ostracism, its centrality is low (Figure 2). The low betweenness index and the small number of direct connections (Table 4) depend on the fact that, as expected, WO as a job characteristic initiates processes, but does not play mediation roles.

## 6 Discussion

In this study, we have demonstrated the validity of the Italian version of the workplace ostracism scale (Ferris et al., 2008). This version proves to be a monodimensional and reliable tool, quite independent of social desirability issues. Moreover, the scale exhibits metric invariance and partial strict invariance across genders and age groups, suggesting that it maintains the same meaning and functioning across the examined groups (the difference regarding intercepts only pertains to items 7 for genders and 2 for age categories; in contrast, the difference regarding residuals only pertains to item 8 for both comparisons). Hypothesis 1 proves to be confirmed. The Italian WOS also has nomological validity: it allowed us to replicate relationships between constructs, that are either included in the JD-R theory or, though not included, observed when testing the theory (data supported Hypotheses 2a–2c, in which WO was conceptualized as a demand).

Overall, our work offers further validation of the job demands-resources theory, in particular of Proposition 2 concerning the effects of demands and resources (Bakker et al., 2023). It also validates the self-determination theory (Deci and Ryan, 2000; Ryan and Deci, 2000, 2017), showing a significant connection between need satisfaction and work engagement (the strongest edge in Figure 2).

Figure 2 highlights that work overload (a demand) is positively and directly related to job autonomy (a resource) and citizenship behaviors. Probably work overload (i.e., time pressure and difficult tasks) functions as a challenge demand, that is, it is perceived by employees as an effortful but rewarding job experience. The distinction between hindrance and challenge job demands, proposed by LePine et al. (2005), is embedded in the JD-R theory (Bakker and Demerouti, 2017). Findings on work overload, therefore, provide further support for this theory.

In this study, based on the JD-R theory (Bakker et al., 2023), workplace ostracism was conceptualized as a job demand, namely as a social aspect of the job that requires sustained cognitive and emotional effort and is, therefore, associated with physiological and psychological costs: a definition coherent with research concerning WO (Howard et al., 2020; Bedi, 2021; Li et al., 2021; Asmita et al., 2024). Future research should support this conceptual proposal. Experiments should be conducted to verify whether manipulated workplace ostracism is related to depression and emotional exhaustion and, through the health impairment process, to low performance and dysfunctional behaviors, such as poor communication and interpersonal conflicts. The inclusion of workplace ostracism in the JD-R theory allows scholars to enhance their knowledge of its effects and identify strategies for its reduction.

Regarding network analysis, which was used in the nomological validation of the WOS, it proved to be a useful tool for detecting the complex system of conceptual relationships included in the JD-R theory. This method, in fact, allowed us to show the centrality of need satisfaction in the processes associated with well-being at work (Hypothesis 3; see the central position of this concept in the graphical representation of the network in Figure 2). The node of basic needs is directly connected to all the other nodes, except for the one of work overload. As to the direct—unexpected—relationships with performance and citizenship behaviors, they may depend on employees reciprocating the satisfaction of their needs with behaviors that are functional to the organization (see the social exchange theory; Gouldner, 1960; Blau, 1964). The two direct links can also be explained considering the conservation of resources (COR) theory (Hobfoll, 1989). According to the COR theory, when people are high in resources (as indicated by high need satisfaction), they engage in more OCBs and higher performance—leading to positive outcomes—to maintain or increase their existing resources (for OCBs, see Spanouli et al., 2024).

Figure 2 shows that citizenship behaviors are connected to numerous nodes. Some of these connections may be bidirectional. For instance, work engagement—the feelings of vigor and dedication to one’s work—may favor citizenship behaviors; citizenship behaviors, in turn, may increase vigor and dedication. Job autonomy—the perception that employees enjoy a certain degree of freedom in performing their tasks—can favor citizenship behaviors (i.e., colleagues are helped, innovation is promoted); these behaviors, in turn, can reinforce the perception that the organization facilitates employees’ autonomy. Future longitudinal, cross-lagged studies should be conducted to test these predictions of bidirectionality. Clearly, network analysis is a useful tool for generating hypotheses of bidirectional causality.

An interesting cluster of job characteristics (WO, social support, and role ambiguity) is represented on the left side of the graph (Figure 2). It suggests potential interventions focused on social support. The aid offered by some colleagues can decrease ostracism actions directed at a target. It can also improve the understanding of company rules, thus limiting the enactment of dysfunctional behaviors, which can induce ostracism (see the negative edge linking social support to role ambiguity in Figure 2). (For a review of interventions aimed to limit workplace ostracism, see the work by Mohammad and Nazir, 2023.) Thus, network analysis may offer guidance for generating intervention proposals.

The current study is not without limitations. First, the nomological validation of the WOS is based on a cross-sectional design, which does not allow causal inferences. Future longitudinal and experimental studies are needed to test the hypothesized associations and the post-hoc associations suggested by our findings. Additionally, we exclusively used self-report measures associated with common method biases (Podsakoff et al., 2003). Future research would benefit from combining different techniques, including observers’ evaluations of job demands, job resources, and citizenship behaviors. However, it should be noted that the application of the partializing technique in network analysis removes from the zero-order correlation between two variables what is common with all the other variables and, therefore, common method factors. To simplify the representation of the network, we combined the three needs into a single measure, thus losing their unique associations with the other variables. Previous research may justify this choice, showing that the satisfaction of each of the three needs is positively related to job resources, such as job autonomy and social support; negatively related to mistreatment; positively related to engagement, task performance, and citizenship behaviors (the relationships of each of the three needs with demands are, instead, less uniform; see the meta-analysis by Van den Broeck et al., 2016). Finally, to simplify the network representation, we did not include emotional exhaustion in the nomological validation of the WOS. Future research should consider this variable to show how workplace ostracism is associated with the impairment process.

In this study, we did not evaluate the convergent and discriminant validity of the Italian WOS. This method of validation would have required the use of scales measuring similar and dissimilar concepts from ostracism. To explore convergent validity of the original WOS, for instance, Ferris et al. (2008) used the concept of undermining, defined as the behavior intended to hinder the ability to establish positive interpersonal relationships and work-related success (Duffy et al., 2002). To explore discriminant validity, they considered the concept of interpersonal justice, namely, the inclination to treat employees with respect and politeness (Colquitt, 2001). Future tests of the Italian WOS should also consider this validation method. Regarding criterion-related validity, that is, the extent to which a construct is associated with variables derived from theory, in the current study, it has been demonstrated by the nomological analysis which is primarily based on the job demands-resources theory.

This study has practical implications. As mentioned above, network analysis highlights that social support from colleagues may be a key variable for containing ostracism episodes. For other constructs, the numerous direct edges of OCB (Figure 2) indicate that several interventions can be used to favor prosocial behaviors. For instance, OCB may be increased by increasing need satisfaction, a finding that can be achieved by working on job resources (e.g., autonomy, Figure 2), favoring positive leader behavior (see Van den Broeck et al., 2016), and encouraging job crafting (see van Wingerden et al., 2017). These interventions can also raise work engagement, a mindset associated with OCB.

To conclude, this study has supported the validity of the Italian version of the WOS. It has also shown the usefulness of applying network analysis to evaluate the JD-R theory; it allowed us, for instance, to show the centrality of need satisfaction in explaining well-being at work (we are not aware of other studies using this method to test the JD-R theory). Finally, the conceptualization of WO as a job demand makes it possible to discover new antecedents, moderators, and consequences of this significant construct.

## Data Availability

The raw data supporting the conclusions of this article will be made available by the authors, without undue reservation.

## Footnotes

^1^Job burnout is a psychological syndrome in response to chronic stressors on the job. The three dimensions of this syndrome are: (a) chronic emotional exhaustion, (b) feelings of cynicism and detachment from one’s job, and (c) a sense of decreased professional efficacy. Exhaustion refers to the perception of being drained of one’s emotional and physical resources. Cynicism represents an overly detached response to various facets of one’s job. The reduced efficacy component includes feelings of incompetence and lack of achievement in one’s work (Schaufeli et al., 1996, 2009; Maslach et al., 2001; see also the definition by the World Health Organization, 2019).

^2^The conclusion that the residual variance is the same for all items except for item 8 is supported by all measures, except for ΔSRMR, which was slightly higher than |0.010|.
